# Mitral valve flattening and parameter mapping for patient-specific valve diagnosis

**DOI:** 10.1007/s11548-019-02114-w

**Published:** 2020-01-18

**Authors:** Nils Lichtenberg, Pepe Eulzer, Gabriele Romano, Andreas Brčić, Matthias Karck, Kai Lawonn, Raffaele De Simone, Sandy Engelhardt

**Affiliations:** 1 Institute for Computational Visualistics, University of Koblenz-Landau, Koblenz, Germany; 2 Department of Cardiac Surgery, Heidelberg University Hospital, Heidelberg, Germany; 3 Department of Anaesthesiology, Heidelberg University Hospital, Heidelberg, Germany; 4 Institute for Computer Science, Friedrich-Schiller-University, Jena, Germany; 5 Working Group Artificial Intelligence in Cardiovascular Medicine, University Hospital Heidelberg, Heidelberg, Germany

**Keywords:** Mitral valve, Quantification, Visualization, Paramterization

## Abstract

**Purpose:**

Intensive planning and analysis from echocardiography are a crucial step before reconstructive surgeries are applied to malfunctioning mitral valves. Volume visualizations of echocardiographic data are often used in clinical routine. However, they lack a clear visualization of the crucial factors for decision making.

**Methods:**

We build upon patient-specific mitral valve surface models segmented from echocardiography that represent the valve’s geometry, but suffer from self-occlusions due to complex 3D shape. We transfer these to 2D maps by unfolding their geometry, resulting in a novel 2D representation that maintains anatomical resemblance to the 3D geometry. It can be visualized together with color mappings and presented to physicians to diagnose the pathology in one gaze without the need for further scene interaction. Furthermore, it facilitates the computation of a *Pathology Score*, which can be used for diagnosis support.

**Results:**

Quality and effectiveness of the proposed methods were evaluated through a user survey conducted with domain experts. We assessed pathology detection accuracy using 3D valve models in comparison with the novel visualizations. Classification accuracy increased by 5.3% across all tested valves and by 10.0% for prolapsed valves. Further, the participants’ understanding of the relation between 3D and 2D views was evaluated. The *Pathology Score* is found to have potential to support discriminating pathologic valves from normal valves.

**Conclusions:**

In summary, our survey shows that pathology detection can be improved in comparison with simple 3D surface visualizations of the mitral valve. The correspondence between the 2D and 3D representations is comprehensible, and color-coded pathophysiological magnitudes further support the clinical assessment.

## Introduction

Surgeries and catheter-based interventions to fix mitral valve (MV) defects are complex and require thorough planning and postoperative evaluation. Transoesophageal echocardiography (TEE) is a standard clinical modality to obtain image data of the MV, which can suffer from multiple and very complex pathologies that alter the geometry of the valve and that ultimately affect their function. In particular, 3D probes allow physicians to obtain an insightful view on the valve. Most clinical workstations offer a direct volume visualization of the captured data. However, they lack the ability to highlight important clinical pathology indicators at a glance. Thus, more enhanced visualization techniques should be added to the available tools for improved clinical assessment and surgical planning.

**Fig. 1:**
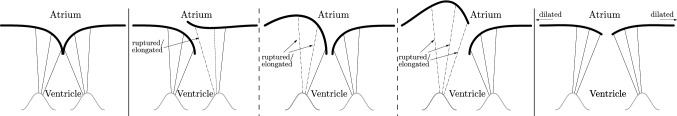
From left to right: a healthy, flail, billowing and prolapsed MV and functional MI during systole. The healthy valve separates the ventricle from the atrium (left). The diseased valves do not close due to ruptured chrodae tendineae (center left to center right). The functional MI prevents closure due to dilation (right)

The MV consists of two leaflets, embedded into the mitral annulus and connected to the papillary muscles by chordae tendineae. The function of the MV is to prevent backflow of blood into the left atrium during systole. Different pathologies can hamper this functionality, resulting in mitral regurgitation (MR). Our approach primarily supports the analysis of prolapsed MVs and valves with functional mitral insufficiency (MI). In prolapsed MVs, which is a degenerative form of MR, the chordae tendineae are either prolonged or ruptured and fail guide the MV leaflets into a closed state during systole. The prolapse can affect either a single leaflet segment or multiple segments, making qualitative and quantitative assessment on 2D TEE data error-prone. There are further variants of the degenerative case, called flail and billowing leaflet. Functional MI is caused by a dilated left ventricle, i.e., the MV leaflets are not large enough to close the valve, causing less coaptation. An illustration of a healthy MV and the pathologies during systole is given in Fig. 1.

The 3D convoluted surfaces of the MV leaflets are difficult to be fully understood on volume rendered TEE image; hence, advanced visualization strategies are a valuable asset. For analysis tasks, flattened representations of anatomical structures are becoming increasingly popular in the domain of medical visualizations. Such 2D representations allow the assessment of a whole object in a single view. Flattening techniques are predominantly projection-based and rely on mesh paramterization. This requires the creation of bijective mappings between a parameter domain in $\mathbb {R}^2$ and a triangulated surface embedded in $\mathbb {R}^3$. In this way, every point in the parameter domain is uniquely associated with a point in the target domain. This is a well-known problem in the computer graphics field, where 2D texture images are mapped to 3D surfaces [1]. A bijective mapping applied to the whole target domain is also referred to as a global mapping, because the whole target domain is considered in the process of mapping to the parameter domain.

In this work, we will transfer the MV models to a disklike topology and apply a global mapping. We propose a flattened view of patient-specific MVs through global paramterization that preserves original structure in terms of tissue area and shape. We use a boundary-free approach that allows us to fix certain landmarks in the parameter space, enhancing overall comparability. This is, for instance, crucial as a basis for the pre- and postoperative comparison of the same patient. As a novel depiction of the MV, the resulting 2D view of a MV should be used in connection with the original 3D representation. Pathophysiological parameters are then mapped to both 2D and 3D views, in order to facilitate the visualization of the MV properties. The performance of this combined representation will be evaluated in a user study. Furthermore, the 3D and 2D representation are utilized to develop an individual score to differentiate pathologic from non-pathologic valves. Our core objectives can be wrapped up as follows:

- Sect. 3.2: A bijective, landmark-based, equiareal mapping between the 3D and 2D representations, which retains the proportion of the valve and preserves spatial context.
- Sect. 3.3: Application of pathophysiological mappings and localization supporting mappings in order to facilitate comprehension of the MV geometry.
- Sect. 3.4: A quantification method based on the 3D and novel 2D representation.
- Sect. 4: A user study that evaluates the performance of our visualization approach w.r.t. the application in a clinical workflow.

In this paper, we build upon our previous publication [9] by providing additional detail on the background, related work and method and conduct a more comprehensive user study, which is then followed by a paramterization-based quantification method.

**Fig. 2:**
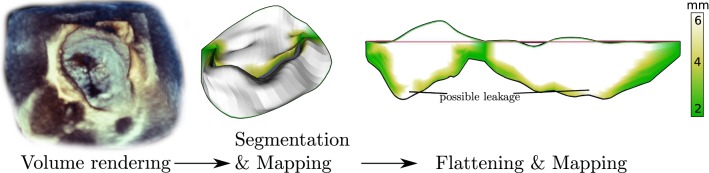
Image-based MV assessment: volume rendering of TEE image allows for initial assessment. More detailed analysis is obtained by means of a MV segmentation. The 2D representation allows for a novel perspective onto the data and new insights. Model-based visualizations are shown with coaptation mapping (cf. Sect. 3.3) and allow thorough evaluation of the data

With our contribution, we envision to augment the existing image-based MV assessment capabilities provided by clinical work stations. An overview of the envisioned work flow is given in Fig. 2. Here, the volume rendering provided by a clinical work station is used for initial assessment of the valve. An extracted 3D model and its 2D projection are then used for further in-depth quantification and decision making. The flattening technique proposed in this paper (Fig. 2, right) builds upon a MV segmentation based on Engelhardt et al. [4] (Fig. 2, center) and provides a novel, supportive view of the MV to enhance diagnosis and therapy decisions. The segmentations used in throughout this paper are manually refined by an expert within a semiautomatic segmentation process. The follow-up work by Eulzer et al. [8] extends the analysis of single time steps to time series of MV models.

## Related work

This section will cover previous work from the mesh paramterization domain and visualization of MV data sets. A general overview of mesh paramterization techniques can be found in [13]. This field is based on early work in graph theory [20] and most commonly applied to computer graphics in purposes of texture mapping [1, 15]. Here, a one-to-one mapping from 3D surfaces to the 2D domain is sought in order to map textures to the surface with minimized or user-controlled distortion. Other approaches can reduce distortion even better, even for arbitrary topology, but do this at the cost of the one-to-one mapping property. Then, bijectivity is only given locally, as in Ray et al. [17].

The visualization of 3D MV models has been applied in the context of MV simulation. For example, Rim et al. [19] studied the effect of leaflet-to-chordae contact interaction and visualized the result as a color map. In a virtual leaflet resection scenario, Rim et al. [18] color-code leaflet stress measurements before and after the virtual resection. Similar depictions of mapped magnitudes can be found in the work by Zhang et al. [21]. While the applied color maps allow to perceive distinct differences of individual data sets, a precise comparison may be hampered due to the convoluted leaflet morphology. Our proposed flattening aims to support such mappings by enabling a clean overview of the MV shape, so that applied maps can be interpreted and compared more precisely and with less cognitive effort. Thus, a comprehensible overview of the MV is not only required in the clinical context, but also in the domain of simulation and modeling.

Flattened depictions have been proposed for the circulatory system, the colon, the brain and the bones. A 2D representation of aortic valve prostheses has been introduced as well [2]. Properties like stent compression were assessed after implantation in order to analyze complication co-occurrences. However, their 2D maps are the result of a cylindrical mapping, i.e., the map always has a rectangular shape and high distortion may occur. Anatomical landmarks are then projected to that map for guidance. We contrast this approach by applying an area-preserving mapping to a surface representing MV tissue. This leads to a 2D representation that more closely follows the input morphology and preserves landmarks. As the MV morphology of individual patients is more variable than that of cylindrical stents, this is a necessary step. A recent state-of-the-art report [14] reviews a variety of medical visualization techniques focused on planar representations. The report covers several approaches that are predominantly tailored to a specific task, which is due to varying requirements and differently shaped input data. To the best of our knowledge, we are the first to contribute to this area with an approach specific for MV models.

## Materials and methods

**Fig. 3:**
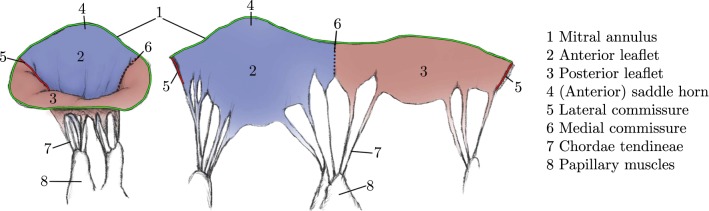
MV similar to depictions in anatomy books with closed (left) and flattened valve (right), cut along the lateral commissure. Important anatomical features are marked

**Fig. 4:**
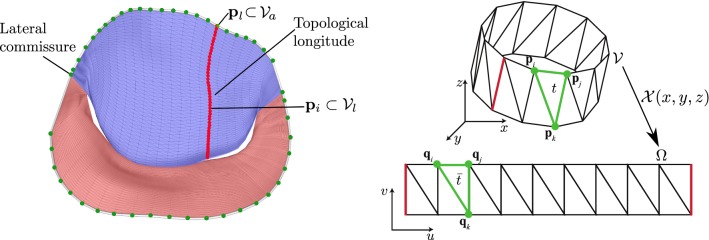
Annulus points $\mathcal {V}_a$ (green), the first one being the point on the lateral commissure (left). Vertices of one topological longitude $\mathcal {V}_l$ (red, left). In the 3D domain, $\mathcal {V}$ consists of points $\mathbf {p}$ and triangles $t \in \mathcal {T}$ and is mapped via $\mathcal {X}$ to $\varOmega$ in the 2D domain with points $\mathbf {q}$ and triangles $\bar{t} \in \bar{\mathcal {T}}$ (right). Red edges depict where the original model is cut to form a disklike topology

We target patient-specific maps and rely on an already existing semiautomatic method to extract MV surface models from 4D ultrasound scans [4], consisting of annulus and leaflets. This segmentation algorithm provides separate triangulations for both MV leaflets and the annulus. Semantic information such as anatomical markers (cf. Fig. 3) is embedded in the representation (cf. Fig. 4, left) and is utilized during the flattening. Chordae tendineae and papillary muscles are not part of these models as TEE images are not capable of depicting these structures reliably.

### Requirements and notation

The mathematical notation of the valve models is described as follows: Each 3D valve mesh consists of vertices $\mathcal {V}\subset \mathbb {R}^3$. A 3D vertex is always denoted as $\mathbf {p}_i = (x_i,y_i,z_i)$ and its counterpart in 2D as $\mathbf {q}_i = (u_i, v_i)$. The points $\mathbf {q}_i$ make up the parameter domain $\varOmega \subset \mathbb {R}^2$, i.e., represent the coordinates of the flattened 2D model. An important subset consists of vertices lying on the annulus $\mathcal {V}_a = \{\mathbf {p}_1, \dots , \mathbf {p}_m \} \subset \mathcal {V}$, where *m* is the number of annulus vertices (cf. Fig. 4, green points, left). Each annulus point is also the first point in a subset $\mathcal {V}_l = \{\mathbf {p}_1, \dots , \mathbf {p}_n \} \subset \mathcal {V}, l \in \{1, \dots , m\}$, which forms a single line from an annulus vertex $(\mathbf {p}_l \subset \mathcal {V}_a) = (\mathbf {p}_1 \subset \mathcal {V}_l)$ to the coaptation (cf. Fig. 4, red points, left). Moreover, the topology of the MV mesh is described by a set of triangles $t \in \mathcal {T}, t = \{\mathbf {p}_i, \mathbf {p}_j, \mathbf {p}_k\}$, with the corresponding set $\bar{t} \in \bar{\mathcal {T}}, \bar{t} = \{\mathbf {q}_i, \mathbf {q}_j, \mathbf {q}_k\}$. An illustration of $\mathcal {V}$ and $\varOmega$ is given in Fig. 4 (right), where the red edge represents the cut along the lateral commissure. The goal of our paramterization will be to find a solution for the function $\mathcal {X}$, to map the points $\mathbf {p}_i \in \mathcal {V}$ to $\mathbf {q}\in \varOmega$. The quality of a flattening technique can be determined through metrics describing the amount of (inevitable) distortions. Usually, paramterization methods focus on preserving either angles (conformal) or area (equiareal) of an input mesh [10]. Fulfillment of both characteristics would result in an isometric or length-preserving paramterization. It is desirable that a 2D view of the MV is close to isometric or at least equiareal. Retaining the proportions of the MV is a primary goal of our method, which should enable the possibility of area and length quantification on the flattened surface. Further, a physician using the 2D view should develop an intuition for its orientation and scale. Hence, apart from minimizing distortions, the 2D view should also target comparability across different data sets through a uniform appearance. Lastly, spatial context should be preserved, i.e., the relation between the 3D and 2D domain should be clear.

The general idea of the proposed flattening algorithm is to cut the MV along its lateral commissure and to unroll it along its diameter. This results in a perspective similar to the valve’s depiction in standard textbooks [3] (cf. Fig. 3, right). In our algorithm, we split the flattening process into three steps:

- Sect. 3.2.1: Annulus paramterization.
- Sect. 3.2.2: Leaflet initialization.
- Sect. 3.2.3: Leaflet relaxation optimization.

The shape of the annulus can give important hints during pathology analysis; therefore, we parameterize it as a curve, independent of the MV leaflets.

**Fig. 5:**
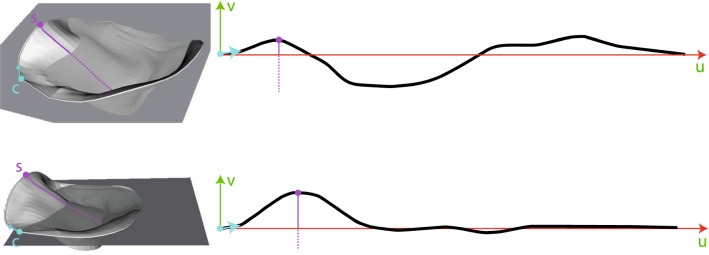
3D MV model and annulus plane (left) constructed by a LS method (top) and the landmark-based method (bottom). Paramterization of annulus (right). Lateral commissure (c) and saddle horn (s) including its iso-*u* line are marked. Please note that the depiction is scaled to fit the parameter range

### Flattening

The annulus parametrization is computed first and its configuration remains unchanged during all further steps. The parametrization preserves the annulus shape and arc length and increases comparability across different data sets. The subsequent steps for the leaflet parametrization are then based on the annulus curve.

#### Annulus paramterization

The annulus’ height is plotted along the *v*-axis and its length along the *u*-axis of the 2D view (cf. Fig. 5). The correspondence of the *u*-axis in 3D is a reference plane through the annulus curve. An intuitive approach to compute the reference plane would be the least-squares (LS) method, resulting in a plane with minimal distances to points on the annulus. However, this approach does not always lead to good results, i.e., the annulus’ height is over- or underestimated (cf. Fig. 5, top). Further, the anterior saddle horn is an important anatomical feature that needs to be accentuated. Height of the saddle horn can be different in dependence of pathologies, e.g., functional MI can be associated with a more planar annular shape [16], while normal or prolapsed valve has a saddle-shaped annulus. Therefore, we employ a landmark-based approach, defining the annulus plane through three points: the two user-defined commissure points on the annulus, which form a natural axis through the MV and the barycenter of the posterior annulus, which usually approximates a planar layout. The annulus curve, with commissure points marked, is provided by the previous semiautomatic segmentation step [4]. This yields an annulus plane $\mathcal {P}_a$ with normal $\mathbf {n}_a$. The annulus parametrization is then obtained as:

1 $$\begin{aligned} \begin{array}{l} v_i = \langle \mathbf {n}_a, (\mathbf {p}_i - \mathrm{proj}(\mathbf {p}_i, \mathcal {P}_a)) \rangle , \\ u_i = {\left\{ \begin{array}{ll} 0 &{} \text {if } i = 1, \\ u_{i-1} + \sqrt{|| \mathbf {p}_i - \mathbf {p}_{i-1} ||^2 - (v_i - v_{i-1})^2} &{} \text {otherwise}, \end{array}\right. } \end{array} \end{aligned}$$

where $\mathrm{proj}(\mathbf {p}, \mathcal {P})$ is the projection of a point $\mathbf {p}$ onto the plane $\mathcal {P}$ and $\langle \cdot ,\cdot \rangle$ denotes the dot product. In this way, the distance of two adjacent points in $\mathcal {V}_l$ is equal to the distance of the corresponding paramterization in $\varOmega$: $||\mathbf {q}_i - \mathbf {q}_{i-1}|| = ||\mathbf {p}_i - \mathbf {p}_{i-1}||$. The resulting 2D view now allows to compare the annulus curve in relation to the *u*-axis, i.e., the location and height of the anterior saddle horn can be assessed in relation to the rest of the curve. Figure 5 compares the result of the landmark-based approach (bottom) with the LS approach (top). It can be observed that the landmark-based method achieves a more intuitive representation of the 3D annulus shape and the anterior saddle horn can be clearly pointed out in the 2D depiction.

**Fig. 6:**
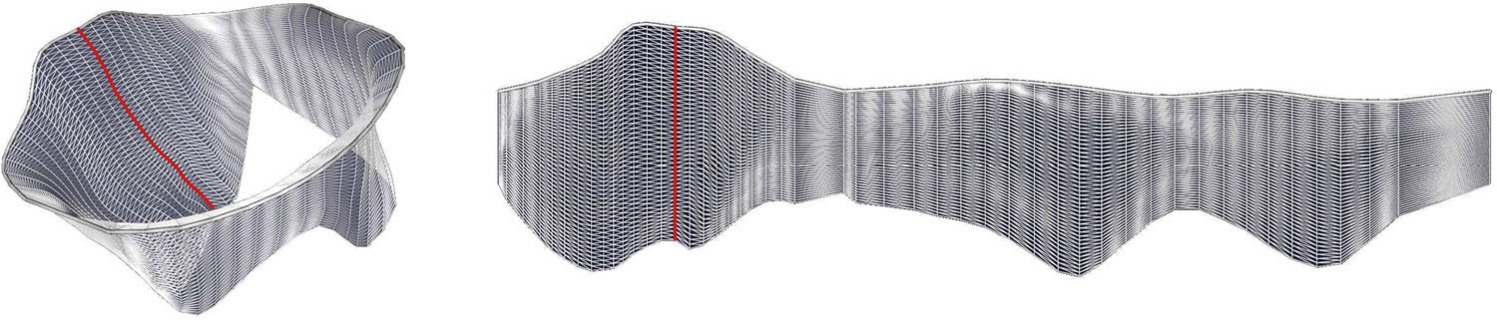
Wireframe of the initial layout of the mapping from 3D (left) to 2D (right) below the already parameterized annulus. An example iso-*u* curve is shown in red

#### Leaflet initialization

The leaflet geometry is placed below the annulus, similar to the appearance of Fig. 3. First, a valid configuration is initialized, i.e., a leaflet layout without self-intersections or triangle flips. We exploit the spline-like lines $\mathcal {V}_l$ which are interpreted as iso-*u* curve approximations and points of each line $\mathcal {V}_l$ are mapped to a shared *u*-coordinate, while the distance between points is preserved in *v*-direction:

2 $$\begin{aligned} \begin{array}{l} u_i = u_1 \qquad \forall i \in [2,n] \\ v_i = v_{i-1} - ||\mathbf {p}_i - \mathbf {p}_{i-1}|| \qquad \forall i \in [2,n], \end{array} \end{aligned}$$

where $\mathbf {q}_1 = (u_1, v_1)$ corresponds to the previously parameterized vertex $\mathbf {p}_1 \in \mathcal {V}_a$ on the annulus. Note that this approach requires the vertices in $\mathcal {V}_l$ to be ordered from annulus to coaptation. An intermediate result of this initialization step is shown in Fig. 6.

#### Leaflet relaxation optimization

As all lines $\mathcal {V}_l$ are now parallel in parameter space, the paramterization does not faithfully reflect the area and morphology of the original 3D valve. Therefore, we optimize toward a more equiareal paramterization. We aim to minimize an energy term describing the distortion amount of the mesh’s edge lengths. If the 3D mesh consists of vertices $\mathbf {p}_i$, corresponding to *uv*-coordinates $\mathbf {q}_i$, and the set $N_i$ contains the indices of all neighbors of $\mathbf {p}_i$, a per-vertex edge length energy can be described as

3 $$\begin{aligned} \mathcal {E}_l = \frac{1}{|N_i|} \sum _{j \in N_i} \frac{||\mathbf {q}_i - \mathbf {q}_j||}{||\mathbf {p}_i - \mathbf {p}_j||} + \frac{||\mathbf {p}_i - \mathbf {p}_j||}{||\mathbf {q}_i - \mathbf {q}_j||}. \end{aligned}$$

Note that this energy reaches its minimum $\mathcal {E}_l = 2$, if and only if all observed edge lengths in the 3D mesh are equal to their counterpart in the parameter domain, and therefore, both fractions evaluate to 1.

We use an iterative Euler method to minimize this energy, modeling the mesh edges in the parameter space $\varOmega$ as a network of springs. The points $\mathbf {q}_i$ are displaced each iteration in the direction of a summed spring force $\mathcal {F}$ calculated based on the model’s edge lengths:

4 $$\begin{aligned} \mathcal {F}_i = \sum _{j \in N_i} k \Big ( ||\mathbf {p}_j - \mathbf {p}_i|| - ||\mathbf {q}_j - \mathbf {q}_i|| \Big ) \frac{\mathbf {q}_j - \mathbf {q}_i}{||\mathbf {q}_j - \mathbf {q}_i||}, \end{aligned}$$

with $k = 1$ being a constant stiffness parameter. A similar method was proposed to simulate MV closure [11]. The above force vector calculation does not take the angles of the edges between the points into account. However, due to our initialization (cf. Eq. 2), the layout of $\varOmega$ is already in an acceptable shape. Further, our main goal is the optimization with area preservation. This is always at the cost of angular distortion. We argue that the area is a more important property, since it can be used to rate the leaflet size or even its stretch over multiple time steps. The angle distortion is therefore omitted during the process, as it would otherwise compete with the area distortion.

**Fig. 7:**
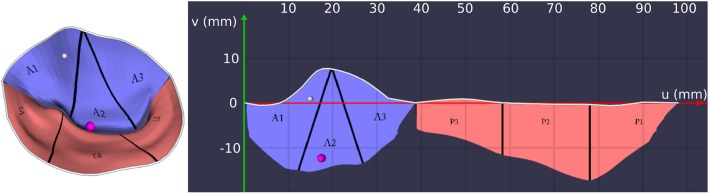
Leaflet subdivision improves 3D–2D correspondence. User-defined points (magenta and white sphere) allow a lookup of points of interest. The grid overlay supports size estimation

**Fig. 8:**
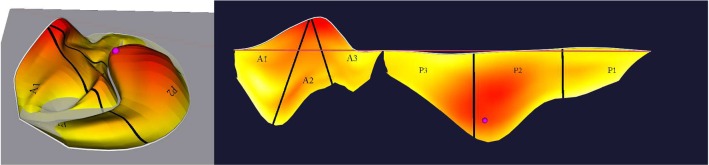
Example of a point (magenta sphere) that represents the most severe prolapse. The color map encodes the signed distance of each point to the annulus plane

### Mappings

After the parameter space has been established, a variety of parameters can be color-mapped onto the 2D and 3D surface. To support a clinical work flow, we address two kinds of mappings:

- *Localization mappings* help to understand the 3D–2D correspondence, by applying the same color or texture pattern in both views.
- *Pathophysiology mappings* display medical information, which can facilitate MV analysis.

We implemented two pathophysiology mappings. The first one shows an approximated coaptation zone, where we used a 2 mm distance threshold between anterior and posterior leaflets to determine the parts of the surface that collide. Please note that the valve segmentations used in this work were obtained in a semiautomatic process and refined by an expert. The coaptation mapping provides therefore another view of the expert’s interpretation of the data. The other mapping is similar to a height map. It marks areas of the MV which are above and below the annulus plane. All mappings are applied to both the 3D and 2D views, which are always rendered side by side. To support localization, we further separate the surfaces into anterior (A1, A2, A3) and posterior leaflet (P1, P2, P3) segments as proposed by Carpentier et al. [3] (cf. Fig. 7). This is a common anatomical definition used in clinical routine. A popular approximation is to subdivide the valve leaflets along the free edge into three equal segments. In our case, the subdivision is implemented based on the parametrization $\varOmega$. For the posterior leaflet, all three segments occupy an equal range of the *u* parameter. The anterior leaflet is split up such that the three segments join at the vertex representing the center of the anterior annulus curve, while at the coaptation, the segments occupy an equal range of the *u* parameter. Figure 7 additionally shows two spheres (magenta and white), placed on the 2D and 3D surface. The spheres can be placed by the user to get a precise feedback of the point correspondence, e.g., to measure the distance of the selected points or to compare local magnitudes derived from the model data. Finally, a grid overlay with 10 mm spacing allows for a quick estimation of the leaflet size.

### 3D–2D-based quantification

The 2D layout of the valve (and also in combination with the 3D structure) enables new ways for valve quantification that represents anatomical measurements, which is detailed in our follow-up work by Eulzer et al. [8]. Here, we give an additional example on how the newly obtained information can be employed for quantification in a sense that we compute a marker for each valve. We propose a *Pathology Score* which indicates a likeliness that a given valve model has a prolapsed morphology. For a MV model with points *i*, the *Pathology Score*$\mathcal {S}_P$ is defined as:

5 $$\begin{aligned} \mathcal {S}_P = \sum _i \bigg [\max (0, \langle \mathbf {n}_i, \mathbf {p}_i - \mathrm{proj}(\mathbf {p}_i, \mathcal {P}_a)\rangle ) \cdot |\min (0, v_i)|\bigg ]. \end{aligned}$$

With this, only points *i* whose 2D parameters $v_i$ are negative and whose signed distances to the annulus plane $\mathcal {P}_a$ are positive are considered. The resultant sum is an abstract score, where points further away from the annulus (i.e., with a lower parameter *v*) are taken into account with a larger weight. An example is given in Fig. 8, where a point that maximizes the individual addend is depicted. In this figure, there are points at the saddle horn that have a higher distance to the annulus plane, but are not located in the negative *v*-space of the 2D paramterization. Thus, the method can be used to detect an appropriate point to represent the area of most severe prolapse.

## Evaluation

The evaluation of our technique is split into a technical and practical part. The technical part covers the performance of the paramterization algorithm and the proposed *Pathology Score* and the practical part covers the evaluation of our visualization approach in the context of MV assessment user study.

**Fig. 9:**
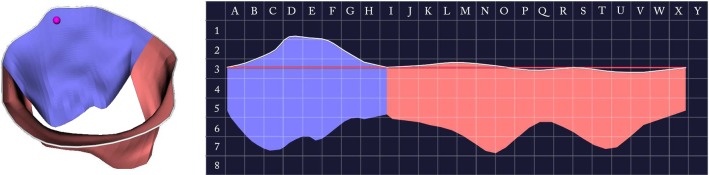
Localization task without localization mapping. In this example, the correct corresponding grid cell for the point shown in 3D would be *D2*

### Paramterization evaluation

To evaluate the employed optimization step, we performed measurements concerning area, angle and edge length deformation (Eq. 3) using 50 MV models and compared the results before and after the spring relaxation method. The area distortion was measured as:

6 $$\begin{aligned} \mathcal {E}_{A,i} = \frac{A(t_i)}{A(\bar{t}_i)} + \frac{A(\bar{t}_i)}{A(t_i)}, \end{aligned}$$

where *A*(*t*) is the area of a triangle in 3D space and $A(\bar{t})$ the area of the affiliated triangle in 2D space. Summing up both the ratio of values and the inverse ratio assures that the result reflects both variants of area distortion: shrinkage and enlargement. The angle distortion is computed according to the formulation found in [12]:

7 $$\begin{aligned} \mathcal {E}_{M,i} = \frac{\cot \alpha |a|^2 + \cot \beta |b|^2 + \cot \gamma |c|^2 }{2 A(t_i)}, \end{aligned}$$

where *a*, *b* and *c* are the edge lengths of a triangle $t_i$ and $\alpha , \beta$ and $\gamma$ are the interior angles of $\bar{t_i}$, opposite of the respective edges. The minimum of $\mathcal {E}_M = 2$ is reached if *t* and $\bar{t}$ have equal angle-to-edge proportions.

### Pathology Score

We investigate whether the *Pathology Score*$\mathcal {S}_P$ can be used for facilitation of MV analysis. First of all, $\mathcal {S}_P$ is compared with an expert assessment regarding the general state of a valve: Is it healthy or pathologic? Further, we asked a heart surgeon to provide additional detail on pathologic valves, by adding information on whether the valve has a *flail*, *prolapsed* or *billowing* leaflet. The above categories are different forms of pathologic valves that have varying causes and require individual treatment. The rating is compared with $\mathcal {S}_P$ in order to find a pattern in the data.

### User study

We assessed the capabilities of the proposed visualization in a user study conducted with one visualization expert, three cardiac surgeons and one anesthetist. After an introductory video, subjects were given a point localization task, where they were asked to mark corresponding points in the 3D and 2D views. The tasks were given with the leaflet segments shown as in Fig. 7 and without the segments. In each task, a point was given in either the 3D or 2D view and the 2D or 3D view was augmented with a grid layout. Subjects were then asked to point out the cell in the 2D grid that corresponds to the marked point in 3D (cf. Fig. 9).

**Fig. 10:**
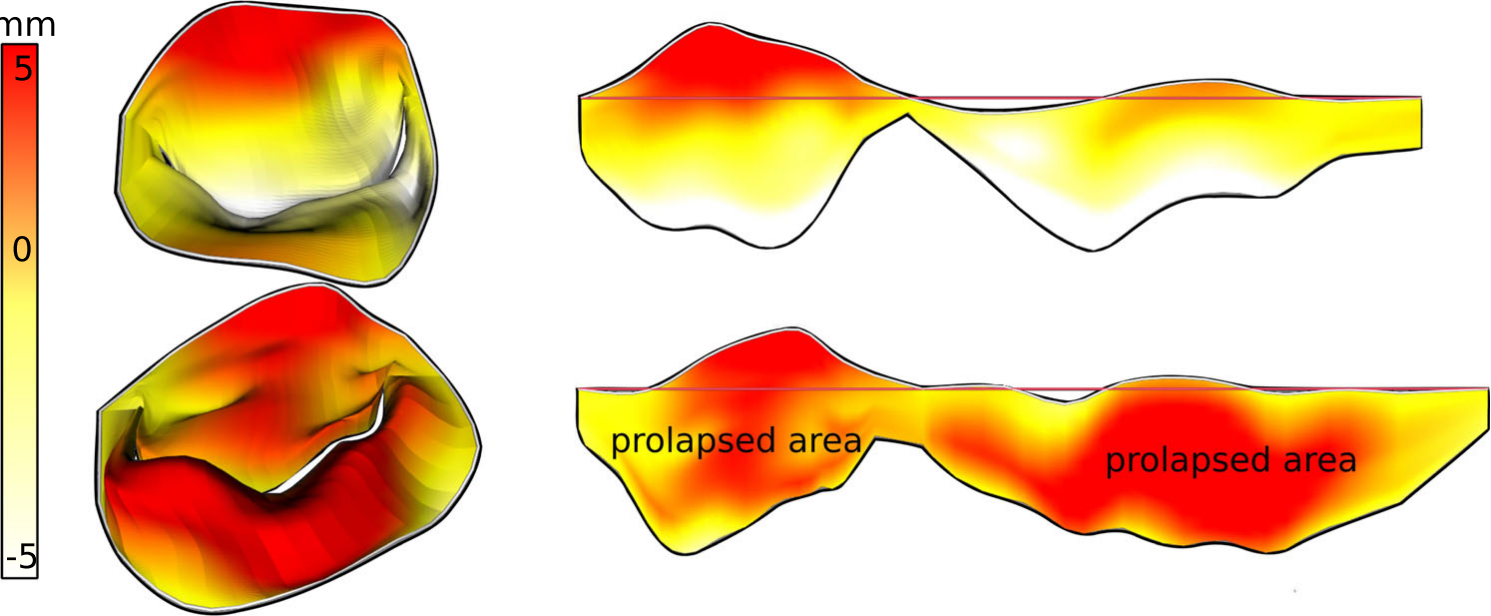
Combined 3D (left) and 2D (right) views with pathophysiological mapping for a healthy valve (top) and prolapsed valve (bottom). The depicted prolapse (height) mapping shows the signed distance of a leaflet point to the annulus plane

The next task was designed to simulate clinical decision making. Within an interactive prototype of our implementation, participants were subsequently shown 20 MV models, each in two alternating formats, resulting in 40 tasks: Half of the tasks displayed models only in 3D (without color-coding) and the other half in a combined 3D–2D view (with color-coding). In the latter, participants could access the coaptation and the height map (cf. Fig. 10). Participants were asked to assign each valve to a category: normal, prolapsed or functional mitral regurgitation. The tasks were presented in a randomized order, making direct comparison possible. Participants were further instructed to mark their confidence in their classification on a Likert scale from one (not confident) to five (very confident).

After the hands-on part of the evaluation, participants filled out a questionnaire, which mainly consisted of providing approval ratings for specific statements concerning the 2D view. Again, a Likert scale from one (strongly disagree) to five (strongly agree) was given. Each participant had to rate the following statements:

*C1*: The correspondence between the 3D and 2D views is clear.
*C2*: Different valves result in distinguishable 2D view.
*C3*: Pathologies of the MV are easier to identify when the 2D view is provided.
*C4*: Analysis of the MV is facilitated through the 2D view.

## Results

Evaluation of the relaxation step showed that we were able to optimize the average edge length energy $\mathcal {E}_l$ from $2+0.78$ before the relaxation to $2+0.07$ afterward. The area distortion dropped from $2+1.9$ to $2+0.2$. This is at the cost of the angular distortion, which was not addressed in the optimization formulation and remained almost equal at a value of $2+1.4$. With this, the goal of achieving an equiareal mapping of the MV is met to a satisfying amount. Remember that the minimum of each magnitude is 2 and that distortion is inevitable due to the transformation from the complex MV morphology to a planar representation.

The point localization task revealed that the 3D–2D correspondence can be well understood, especially with the leaflet segments being displayed. Without leaflet segments, the average distance of the correctly mapped 2D point to the point selected by the subject (measured from correct grid cell center to selected grid cell center) was approximately 4 mm (or 0.74 cells). The average distance with leaflet segments on was zero, w.r.t. the grid cell size. This means that all subjects were able to select the correct grid cell in every task with segments displayed. Note that diagnosis is usually done per leaflet segment. Thus, we consider the above precision as sufficient.

**Table 1:** Average of the pathology identification task per participant *P* regarding accuracy $A_i$, confidence $C_i$ and time $T_i$ for 3D only view ($i=0$) and 3D–2D combination ($i=1$)

*P*	$A_0$ (%)	$A_1$ (%)	$C_0$	$C_1$	$T_0$	$T_1$
1	78.9	84.2	3.89	4.16	11.5s	15.4s
2	68.4	73.7	4.37	4.53	14.0s	15.7s
3	78.9	89.5	4.58	4.84	15.9s	12.6s
4	84.2	73.7	3.00	3.00	13.9s	13.2s
5	84.2	100	4.53	4.68	15.9s	16.0s
Total	78.8	84.2	4.07	4.24	14.3s	14.6s

Two examples of the resulting visualization are shown in Figs. 2 (right) and 10. The visual analysis of the 2D maps provide a much more detailed understanding of the pathology: In the 3D view, it is not clearly visible whether the valve fully closes or not. In contrast to that, the 2D coaptation view (Fig. 2, right) indicates a part where the leaflets do not touch. Beyond that, the height map (Fig. 10) illustrates the extent of prolapsing areas very well, i.e., tissue which surpasses the annulus plane in systole is marked in red.

The pathology assessment survey showed an increase in the pathology detection rate in 4 out of 5 participants when they had access to the 3D surface and the flattened MV, including the coaptation and prolapse color maps (cf. Table 1). A total average of 5.3% increased accuracy was measured. Most noticeably, global detection accuracy for prolapsed valves rose from 85 to 95%. As the observed data are not normally distributed, we conducted a Friedman’s ANOVA test, which resulted in $\chi ^2(2) = 0.88$ and $p = 0.348$. Thus, statistical significance is not given with this study. However, we have to point out that the significance theoretically increases with a higher number of subjects at equal performance. The time required for one classification averaged at about 14 s, regardless of the view mode. Participants were slightly more confident in their decisions when using the combined view. Average confidence (discrete scale from 1 to 5) rose from 4.07 to 4.24. When making incorrect classifications, participants reported an average confidence of 3.53 in both view modes. For correct classifications, average confidence increased from 4.08 (3D only) to 4.28 (3D–2D). The scores obtained for the four categories of the questionnaire underline that the subjects are in favor of the proposed visualization technique and see the potential for improved MV assessment. The average scores from 1 (strongly disagree) to 5 (strongly agree) were: (*C1*, 4.9), (*C2*, 4.2), (*C3*, 4.6) and (*C4*, 4.6).

**Fig. 11:**
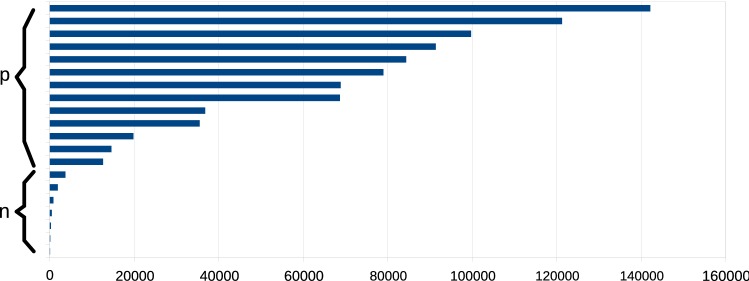
*Pathology Score*$\mathcal {S}_P$ for individual valves on the horizontal axis. The vertical axis labels the valves with non-pathologic (*n*) and pathologic (*p*) morphology

Using combined information from the 2D and 3D representation, we assigned the *Pathology Score*$\mathcal {S}_P$ to each valve. As shown in Fig. 11, healthy valves obtain a very low score, while heavily pathologic valves reach very high scores. Thus, the score can be used as an initial hint that an affected valve is present and may be incorporated for automatic processes. The division into *flail* (*F*), *prolapsed* (*P*) and *billowing* (*B*) leaflets showed that only three anterior leaflets were pathologic in our data set. These are therefore disregarded due to the too small sample size. The diseased valves were, however, all affected at the posterior leaflet ($|F| = 3$, $|P| = 6$, $|B| = 3$; one pathologic valve was ischemic and has not been considered here, as the cause of the disease is not directly associated with the leaflet morphology). The mean and standard deviation of the *Prolapse Score* (normalized to a maximal value of 1) are then given for each pathology category: *F* ($\bar{\mathcal {S}}_P = 0.23 \pm 0.15$), *P* ($\bar{\mathcal {S}}_P = 0.50 \pm 0.27$) and *B* ($\bar{\mathcal {S}}_P = 0.80 \pm 0.20$). Since the data are normally distributed, we did an ANOVA test resulting in $F(2, 11) = 3.74$ and $p = 0.065$. Statistical significance is therefore given in the data set. A pair-wise comparison yielded: $F \leftrightarrow P$ ($F(1,8) = 2.08$) and $p = 0.192$; $F \leftrightarrow B$ ($F(1,5) = 11.96$) and $p = 0.025$; $P \leftrightarrow B$ ($F(1,8) = 2.42$) and $p = 0.164$. The results indicate that $\mathcal {S}_P$ differs significantly for flailed and billowed leaflets.

## Discussion

We presented an approach for flattening patient-specific 3D MV models, which results in an expressive 2D depiction across different data sets. The visualization targets clinical MV analysis, a process that appears to benefit from the proposed 2D view with color maps. This is underlined by the increased pathology detection rate measured in our survey, which holds true, especially for prolapsed valves. The coaptation zone can be assessed at a glance, as well as the prolapsed valve area. A landmark-based paramterization of the annulus makes comparison of height deviations possible. Low area and edge length distortions of the leaflet geometry allow size quantification of the flattened MV. The evaluation shows that spatial context is preserved as the domain experts had no difficulties understanding 3D–2D correspondence. Participants claimed pathologies were easier to identify, and MV analysis was facilitated when the 2D view was provided. They pointed out that determining the valve part affected by a prolapse was aided by the flattened representation, even though the study was not particularly designed for this aspect. The *Pathology Score*$\mathcal {S}_P$ showed that the paramterization can be utilized to facilitate quantification methods. A significant difference in $\mathcal {S}_P$ was found for flailed and billowing leaflets, so we consider future work to automate the process of detecting different pathologic morphologies. We still have to point out that the sample size for the presented evaluation is low, but the current results should motivate a broader study.

Our leaflet relaxation optimization (cf. Sect. 3.2.3) treats all parts of the MV equally and does not optimize angular distortion. Hence, the final distortion is predominantly influenced by the leaflet initialization step (cf. Sect. 3.2.2). A future extension may involve a more user-controlled parametrization that favors regions of interest and relocates the distortion to less important regions. This would support the faithful visualization for in-depth analyses.

A possible drawback of our method is its low generalizability. The approach is tailored to the MV, and our implementation relies on the structure of the MV model by Engelhardt et al. [4]. However, our results should motivate the extension to more generic representations of the MV and the incorporation of further controllable parametrization methods, as well as additional clinically relevant magnitudes. Future work includes integration of the flattening visualization into an intraoperative assistance system [7] for mitral valve repair to aid the surgeon *during* the procedure and integration in a patient-specific minimally invasive simulator for surgical training and planning [5, 6].
